# Early Neuromuscular Electrical Stimulation Preserves Muscle Size and Quality and Maintains Systemic Levels of Signaling Mediators of Muscle Growth and Inflammation in Patients with Traumatic Brain Injury: A Randomized Clinical Trial

**DOI:** 10.1155/2023/9335379

**Published:** 2023-07-26

**Authors:** Luciana Vieira, Paulo Eugênio Silva, Priscilla Flavia de Melo, Vinicius Maldaner, Joao Q. Durigan, Rita de Cassia Marqueti, Otavio Nobrega, Sunita Mathur, Chris Burtin, Fabrício Barin, Wilcelly Machado-Silva, Sergio Ramalho, Gaspar R. Chiappa, Nadia Oliveira Gomes, Celso R. F. Carvalho, Graziella F. B. Cipriano, Gerson Cipriano

**Affiliations:** ^1^University of Brasilia, Faculty of Ceilãndia, Sciences and Technologies in Health Program (PPGCTS), Brasília, DF, Brazil; ^2^Physical Therapy Division, Hospital de Base do Distrito Federal, Brasília, DF, Brazil; ^3^Human Movement and Rehabilitation Program, UniEVANGÉLICA, Anápolis, GO, Brazil; ^4^University of Brasilia, Faculty of Ceilãndia, Rehabilitation Sciences Program (PPGCR), Brasília, DF, Brazil; ^5^Medical Sciences Graduate Program (PPGCM), University of Brasilia (UnB), Brasília, DF, Brazil; ^6^School of Rehabilitation Therapy, Queen's University, Kingston, ON, Canada; ^7^Rehabilitation Research Centre, Biomedical Research Institute, Faculty of Rehabilitation Sciences, Hasselt University, Hasselt, Belgium; ^8^School of Medicine, University of Sao Paulo (USP), São Paulo, SP, Brazil

## Abstract

**Objective:**

To investigate the effects of an early neuromuscular electrical stimulation (NMES) protocol on muscle quality and size as well as signaling mediators of muscle growth and systemic inflammation in patients with traumatic brain injury (TBI).

**Design:**

Two-arm, single-blinded, parallel-group, randomized, controlled trial with a blinded assessment. *Setting*. Trauma intensive care unit at a university hospital. *Participants*. Forty consecutive patients on mechanical ventilation (MV) secondary to TBI were prospectively recruited within the first 24 hours following admission. *Interventions*. The intervention group (NMES; *n* = 20) received a daily session of NMES on the rectus femoris muscle for five consecutive days (55 min/each session). The control group (*n* = 20) received usual care. *Main Outcome Measures*. Muscle echogenicity and thickness were evaluated by ultrasonography. A daily blood sample was collected to assess circulating levels of insulin-like growth factor I (IGF-I), inflammatory cytokines, and matrix metalloproteinases (MMP).

**Results:**

Both groups were similar at baseline. A smaller change in muscle echogenicity and thickness (difference between Day 1 and Day 7) was found in the control group compared to the NMES group (29.9 ± 2.1 vs. 3.0 ± 1.2, *p* < 0.001; −0.79 ± 0.12 vs. −0.01 ± 0.06, *p* < 0.001, respectively). Circulating levels of IGF-I, pro-inflammatory cytokines (IFN-y), and MMP were similar between groups.

**Conclusion:**

An early NMES protocol can preserve muscle size and quality and maintain systemic levels of signaling mediators of muscle growth and inflammation in patients with TBI. This trial is registered with https://www.ensaiosclinicos.gov.br under number RBR-2db.

## 1. Introduction

Traumatic brain injury (TBI) is a notable cause of morbidity and mortality [1, 2]. Advances in intensive care have decreased the mortality rate [3], but survivors still face substantial functional impairment that exerts a negative impact on quality of life [4]. However, certain patients cannot participate in active exercise in the first days in the intensive care unit (ICU). Therefore, therapeutic methods are required to attenuate muscle atrophy as a consequence of prolonged bed rest in sedated or non-cooperative patients [5].

Neuromuscular electrical stimulation (NMES) is an effective, safe, low-cost tool for early rehabilitation in critically ill patients [6]. However, the benefit of this therapeutic modality in modulating signaling mediators of muscle growth and systemic inflammation remains inconclusive [7]. An NMES protocol initiated seven days after admission to the ICU reduced muscle atrophy during the intervention but did not restore muscle loss that occurred in the first week of ICU stay [8]. Some studies have indicated that early NMES intervention leads to no changes in muscle mass in ICU patients, whereas others studies have shown that muscle hypertrophy occurs when NMES is initiated 14 days after admission [9, 10]. Thus, the best moment for initiating an NMES protocol in the ICU remains largely unknown. The protective effect of NMES from muscle fiber catabolism in ICU patients seems to be related to multiple factors, including clinical characteristics and electrically evoked muscle force [2].

Muscle biopsy is the gold-standard method for diagnosing diseases involving muscle tissue but has some risks, such as bruising, discomfort, and prolonged bleeding at the biopsy site [7, 9, 11], which reduces its clinical use. B-mode ultrasonography has been widely used in clinical practice to assess muscle wasting in ICU patients [12–14] due to its non-invasive and convenient features. Graded echogenicity has been shown to correlate with pathologic findings in muscle biopsies [15]. The loss in muscle thickness, denominated muscle wasting, is associated with weakness that increases the mortality rate in ICU patients [14, 16]. Additionally, it has been demonstrated that an increase in muscle echogenicity (meaning muscles are less dense and fibers are smaller) in ICU patients has been associated with reduced functional capacity [16] and performance during volitional tests [14].

Changes in muscle echogenicity may result from a disruption in the muscle architecture on the cellular level. A recent study used muscle biopsy to identify sequential changes. Edema, neutrophil polymorphs, and fibrin dominated the early phase (Days 1–3) of critical illness, whereas macrophage-predominant cellular fasciitis occurred in the late phase (Days 7–10). An increase in ultrasound muscle echogenicity predicted these changes during the first week in critically ill patients [17].

Most individuals with TBI require prolonged bedrest, mechanical ventilation, and sedation, all of which independently induce muscle atrophy [18]. A persistent increase in inflammatory cytokine levels [19] and a reduction in the secretion of insulin-like growth factor I (IGF-I) lead to worse muscle damage. Both stimuli cause protein synthesis and stimulate protein breakdown, leading to further skeletal muscle atrophy [20]. Inflammatory cytokines play a key role in matrix metalloproteinase (MMP) regulation. MMP-2 acts in the extracellular matrix during muscle growth and development as well as during the muscle repair process [21], whereas MMP-9 reflects a systemic inflammatory state [22].

We hypothesize that the acute effect of NMES in patients with TBI could increase IGF-I levels and minimize muscle catabolism and inflammatory responses. Therefore, the aim of the present study was to investigate the effects of an early NMES protocol on muscle quality and size as well as signaling mediators of muscle growth and systemic inflammation in patients with TBI.

## 2. Methods

### 2.1. Study Design

A randomized controlled, single-blinded trial was conducted at the ICU of a level I trauma center. This study received approval from the local institutional review board (certificate number: 19036013.8.0000.5553), and the clinical trial was registered at https://www.ensaiosclinicos.gov.br under number RBR-2dbzdy. There was no industry support or involvement in the trial.

### 2.2. Protocol Overview

Patients with TBI were assessed for eligibility within the first 24 hours after hospital admission and randomized to either the control or NMES (daily electrical stimulation protocol for five consecutive days) group. Ultrasound measurements were taken of muscle echogenicity and thickness of the rectus femoris muscle. Muscle growth and systemic inflammation were investigated using the following biomarkers: plasma levels of IGF-I, plasma cytokines (IL-2, IL-4, IL-6, IL-10, TNF-*α*, and IFN-y), and matrix metalloproteinases (MMP-2 and MMP-9).

### 2.3. Randomization and Allocation Concealment

Consecutive participants were admitted after written informed consent was obtained from their legal representatives, since the patients were intubated and sedated upon enrollment. Computer-generated randomization lists were created using a website platform (https://www.random.org), which sequentially distributed the patients into the control and NMES groups at a 1 : 1 ratio. A researcher (VM) prepared opaque, sealed envelopes to conceal the allocation. The envelopes were sequentially opened only after participants' details were recorded.

### 2.4. Blinding

Two blinded researchers (LV and PFM) performed all ultrasonography assessments and collected clinical data from each participant's electronic medical record. Another blinded researcher (FB) performed the blood analyses, and one of the blind assessors (LV) also performed all data processing and statistical analysis.

### 2.5. Study Population

All patients with TBI were assessed for eligibility within the first 24 hours after admission to hospital. Adults older than 18 years of age on invasive mechanical ventilation and expected to stay on prolonged mechanical ventilation based on the assessment of a senior physician upon admission were included [23].

The exclusion criteria were pregnancy, stroke, previous neuromuscular disease, suspicion of brain death, lower limb amputation, extremity fracture, or skin lesions that precluded the ultrasound evaluation and NMES.

### 2.6. Interventions

#### 2.6.1. Usual Care

The patients in both groups received usual care consisting of respiratory physiotherapy and progressive rehabilitation twice a day (morning and afternoon) based on the recommendations of the Brazilian Association of Intensive Care Medicine for critically ill adult patients [24]. This protocol establishes five activity levels, starting with passive upper and lower limb mobilization, static stretching, and joint proprioception stimulation (Level 1). Active extremity movements are performed when the patient becomes conscious (Level 2). The progression of activity depends on the patient's ability to move the upper limbs against gravity (Level 3), move the lower limbs against gravity (Level 4), and stand up without assistance (Level 5) as published elsewhere [25].

#### 2.6.2. Neuromuscular Electrical Stimulation

The NMES group received one daily session of electrical stimulation in the afternoon for five consecutive days [26]. NMES was conducted after the daily ultrasound evaluation and collection of the blood sample in the morning. All patients were screened for clinical stability before each NMES session. The session was postponed if patient had any of the following within three hours before the session: received neuromuscular blocker infusion or had documented acidosis (pH of arterial blood gas <7.25), hypertension or hypotension (mean arterial pressure <60 mmHg or >120 mmHg), high intracranial pressure (ICP) (>20 mmHg), or signs of clinical instability (e.g., temperature <34°C or >38°C, platelet count <20000/mm^3^).

NMES settings were based on previous protocols [23, 26, 27]. NMES was implemented simultaneously on the quadriceps muscles of both lower extremities. After shaving and cleaning the skin, four self-adhesive rectangular electrodes (90 × 50 mm; MultiStick®, Axelgaard Manufacturing Co, Ltd, Fallbrook, CA, USA) were placed over the motor points of the rectus femoris muscles of both legs following the method proposed by Botter et al. [28].

Electrode position was marked daily with an indelible marker to maintain the same location for each session. The positions were also marked in the sham NMES group to ensure the blinding of the examiners. The Dualpex 071 stimulator (Quark Medical®, Piracicaba, SP, Brazil) delivered a biphasic, symmetric, rectangular-wave pulse at a frequency of 50 Hz, pulse duration of 400 microseconds (*μ*s), and a duty cycle of six seconds on (including one-second rise time and one-second fall time) and 12 seconds off at intensities able to evoke maximum muscle contraction type 5 according to classification proposed by Segers et al. [29]. Current intensity was increased during the session each time that the quality of the electrically evoked contraction decreased. Session duration was 55 minutes and included 45 minutes of treatment (150 contractions), with five minutes of warm-up and recovery at lower intensities.

### 2.7. Outcomes

The primary outcomes were muscle echogenicity and thickness assessed by ultrasound. The secondary outcomes were signaling mediators of muscle growth and systemic inflammation using plasma levels of IGF-I, cytokines, and MMPs. Clinical variables were analyzed as exploratory outcomes and baseline characteristics.

#### 2.7.1. Muscle Echogenicity and Thickness

Serial ultrasound measurements of muscle echogenicity and thickness of the rectus femoris muscle were obtained daily in the afternoon period during the first five days of hospitalization. All sonograms were acquired with a standardized protocol of transducer placement, anatomic landmarks, and patient position with a SonoSite M-Turbo® portable ultrasound device (Sonosite, Inc., Bothell, WA, USA) equipped with a high-frequency linear array transducer (L38xi) (bandwidth: 10−5 MHz; maximum scan depth: 9 cm).

Two different examiners (LV and PFM) performed the ultrasound measures. The intraclass correlation coefficient for interrater reliability determined in a pilot study with trauma patients was 0.96. The coefficient of variation between different assessors was less than 2.3% (1.4) AU for muscle echogenicity and less than 2.8% (0.08) cm for muscle thickness.

Sonographic settings (frequency, depth, gain, and compression) were kept constant between patients and across all time points. The patients were assessed in the supine position with the knee in passive extension and neutral rotation, as described elsewhere [30]. A water-soluble transmission gel was applied to the ultrasound probe to enable acoustic contact without depressing the skin surface. Three anterior images were obtained from the right leg of the patient at a depth of 5.9 cm with the transducer placed on the anterior surface of the thigh perpendicular to the long axis at 50% of the distance from the anterior superior iliac spine to the superior patellar border along the midline of the thigh. This specific orientation of the probe was chosen because it was the only one that could be standardized without the need for additional equipment (such as probe supports) in a clinical setting.

All image measurements were performed in triplicate using the ImageJ software (NIH, Bethesda, MD, USA), and average values were used in the analysis [31]. Muscle echogenicity was quantified using average echogenicity of the rectus femoris muscle [32]. A standard square area of 2 × 2 cm was used to determine the region of interest. If the defined 2 × 2 cm measurement was found to be larger than the cross section of the muscle, the largest possible square that fit within the anatomic boundaries of the muscle was used [33]. Grayscale was calculated using the histogram function, expressed as a value between 0 (black) and 255 (white) and reported in arbitrary units (AU). Muscle thickness was evaluated by rectus femoris thickness, which was defined as the maximum distance between the subcutaneous tissue and surface of the femur and expressed in centimeters (cm) [32, 33].

#### 2.7.2. Blood Collection and Biochemical Analyses

Biomarkers of muscle growth and systemic inflammation were assessed by measuring circulating levels of IGF-I, plasma cytokines (IL-2, IL-4, IL-6, IL-10, TNF-*α*, and IFN-y), and matrix metalloproteinases (MMP-2 and MMP-9) according to previous publication from our group [34]. Arterial blood samples were collected daily from an arterial line or radial artery puncture in tubes containing EDTA. The tubes were centrifuged at 1000 × *g* at 4°C for 10 minutes with 2500 rotations per minute (centrifuga, MPW-350R, MPW Med. Instruments, Warsaw, Poland). Aliquots of plasma were stored directly at −20°C until further analysis by a blinded examiner (FB). IGF-I levels were measured using the Quantikine® ELISA Human IGF-I Immunoassay kit (R&D Systems, Inc., Minneapolis, MN, USA), with all analyses for the same individual performed in the same plate.

All calibration curves had a coefficient of determination of 0.98 or higher. Intra and interassay coefficients of variation were <5%. Each test detection threshold was determined experimentally at 5 ng/ml. Serum cytokine levels were determined with a commercially available multiplexed flow cytometry method, using a set of bead-based immunoassays manufactured by BD Biosciences (CBA, San Diego, CA, USA) with lower detection limits determined experimentally: 0.01 pg/ml for TNF-*α*, 0.2 pg/ml for IFN-y, 1.0 pg/ml for IL-2, IL-4, and IL-10, and 1.5 pg/ml for IL-6. All assays were carried out according to the manufacturer's protocol.

MMP-2 and MMP-9 gelatinolytic activities were measured by zymography. This is an electrophoretic method for measuring proteolytic activity that uses a sodium dodecyl sulfate (SDS-PAGE) gel impregnated with a protein substrate, which is degraded by the proteases resolved during the incubation period [34, 35]. Samples containing 0.5 *μ*L of plasma were added to 0.5 *μ*L of SDS (8%) (v:v). The mixtures were then added to 10 *μ*L of the buffer without *β*-mercaptoethanol-containing SDS (20%). Samples were resolved by electrophoresis in a polyacrylamide gel containing 10% SDS and gelatin at a final concentration of 1 mg/ml.

After electrophoresis, the gels were washed twice for 20 minutes in 2.5% Triton X-100 to remove the SDS. The gels were incubated in buffer substrate (50 mM Tris-HCl, pH 8.0, 5 mM of CaCl_2_, and 0.02% NaN_3_) at 37°C for 20 hours. The gels were stained with Coomassie brilliant blue for 1.5 h and destained with acetic acid : methanol : water (1 : 4 : 5) for visualization of the activity bands. Gelatinolytic activity was visualized as clear bands in the stained gel. Densitometric quantitative analysis of the MMP protein bands was performed using the ImageMaster 2D Platinum v7.0 equipment (GE Healthcare, Uppsala, Sweden).

#### 2.7.3. Clinical Outcomes

The clinical outcomes were days on sedation, days on invasive mechanical ventilation, number of ventilator-free days at 28 days, length of stay in the ICU and hospital, and seven-day and 28-day mortality.

### 2.8. Sample Size and Statistical Analysis

The sample size was calculated using rectus femoris muscle thickness as the primary outcome (G^*∗*^ power version 3.1.4, Franz, Universitat Kiel, Germany). According to (2) a pilot study with critically ill patients, we estimated a difference of −0.33 vs. −0.49 mm in muscle thickness with a standard deviation (SD) of 0.12 in each group. Considering a study power of 80%, a 95% significance level, and a sample size ratio of 1 : 1 (control and NMES groups), an estimated minimum of 10 patients was needed per group. Seventy-five patients were analyzed for eligibility, and 40 were enrolled (20 per group), enabling possible dropouts during the intervention period while still maintaining the minimum sample size (12) [36].

Data were assessed for normality using the *Shapiro–Wilk* test and reported as mean ± standard deviation or median (percentile 25–75), as appropriate. Statistical significance was indicated by *p*  < 0.05. Clinical outcomes were compared between the control and NMES groups using the unpaired *t*-test for continuous variables and the chi-squared test for the dichotomous variable. Reported *p* values refer to changes between groups and across time evaluated by two-way repeated-measures ANOVA with the *Bonferonni* post hoc test or *Friedman* test, as appropriate (SPSS version 21, IBM Corporation, Armonk, NY). Changes in muscle echogenicity and thickness from baseline to Day 7 were assessed using unadjusted and adjusted regression analysis with the aid of the STATA 13 statistical package.

## 3. Results

### 3.1. Patients

**Figure 1:**
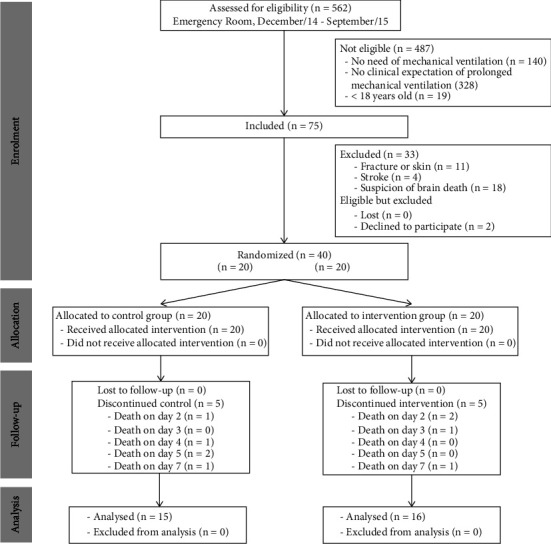
CONSORT flow diagram of patient enrollment, allocation, follow-up, and analysis.

Between January and September 2015, 75 patients met the inclusion criteria at the trauma center of the emergency room at a public hospital. Thirty-three were excluded due to fracture or skin lesions (*n* = 11), stroke (*n* = 4), or suspicion of brain death (*n* = 18), and two families did not authorize participation. Thus, 40 patients were randomly assigned: 20 were allocated to the intervention group (NMES) and 20 to the control group. The CONSORT flow diagram of the study is presented in Figure 1. Losses for the primary outcome occurred only due to patient death in the control (*n* = 5) and intervention (*n* = 5) groups. Baseline characteristics, such as age, sex, and results of the Acute Physiology and Chronic Health Evaluation II (APACHE II), Abbreviated Injury Scale (AIS), and Injury Severity Score (ISS), were similar between groups (Table 1).

In the first week of hospitalization, sepsis occurred in 85% of the control group and 80% of the NMES group (septic shock: 45% and 55%, respectively). Cumulative fluid balance over seven days was positive in both groups: 8.5 ± 6.9 L in control group and 8.2 ± 4.2 L in NMES group (MD [95% CI]: 0.27 [−4.1; 4.7], *P* = 0.89). Mean duration on sedation was 7.6 ± 4.7 days in the control group and 8.8.6 ± 4.9 days in the NMES group (MD [95% CI]: −1.2 [−4.40; 1.91], *P* = 0.42). These data are summarized in Table 2.

### 3.2. Intervention

During the seven days of the study protocol, all patients in both groups were on Level 1 (unconscious) or Level 2 (conscious but unable to move their upper limbs against gravity) of the usual care mobilization protocol, meaning that the patients were unable to cooperate with active exercise in the first week of hospitalization. The NMES protocol consisted of five 55-minute sessions, with a total stimulation period of 275 min per week (750 contractions). No session was omitted due to the prescreening safety criteria, and no adverse effects were observed during the five sessions. Visible muscle contraction was ensured in all sessions.

### 3.3. Outcomes

#### 3.3.1. Muscle Echogenicity and Thickness

Based on previous studies in critical care settings, sonographic images were acquired in less than 10 minutes. Fifteen (75%) individuals in the control group and sixteen (80%) in the NMES group survived after seven days of hospital admission. Losses of ultrasound assessments occurred only due to patient death.

**Figure 2:**
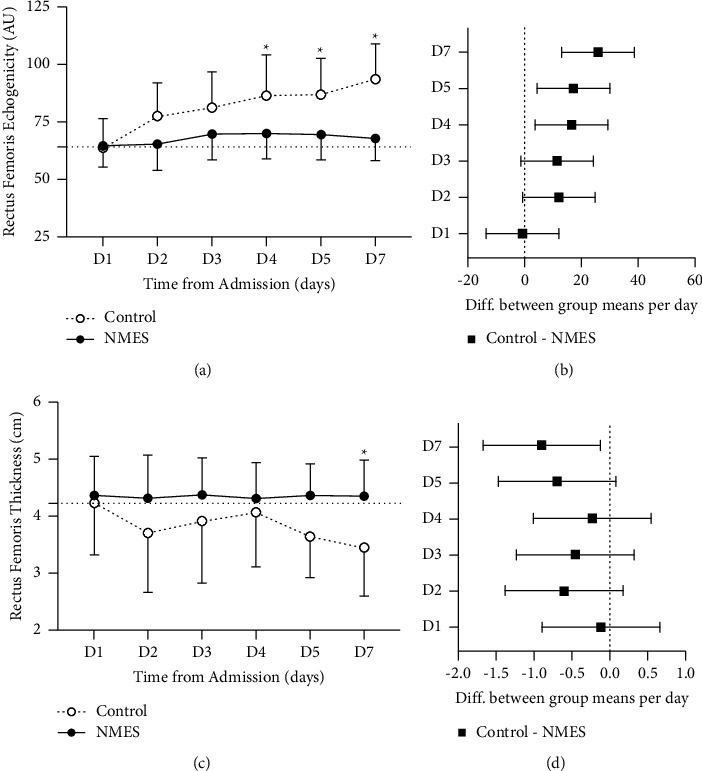
Muscle echogenicity and thickness measured by ultrasound from Day 1 to Day 7 after hospital admission in critically ill trauma patients. (a) Muscle echogenicity. (b) Muscle echogenicity diff. in means per day (D1 to D7) between groups (control vs. NMES). (c) Muscle thickness. (d) Muscle thickness diff. in means per day (D1 to D7) between groups (control vs. NMES). ^*∗*^*p* value <0.05 assessed by two-way ANOVA. Control: control group; NMES: neuromuscular electrical stimulation group.

In the first week of hospitalization, a smaller change in muscle echogenicity (difference between Day 1 to Day 7) occurred in the control group compared to the NMES group (29.9 ± 2.1 vs. 3.0 ± 1.2, respectively; *P*  < 0.001) (Table 3). This change began on Day 4 (Figures 2(a) and 2(b)). Unadjusted delta was 26.9 ([21.98; 31.78], *P*  < 0.001), whereas delta adjusted for baseline was 26.70 ([21.51; 31.82], *P*  < 0.001).

Muscle thickness decreased in the control group (difference between Day 1 to Day 7) and was preserved in the NMES group (−0.79 ± 0.12 vs. −0.01 ± 0.06, respectively, *P*  < 0.001) (Table 3). This change occurred only on Day 7 (Figures 2(c) and 2(d)). Unadjusted muscle thickness delta assessed by regression analysis was −0.78 ([−1.05; −0.51], *P*  < 0.001), whereas delta adjusted for baseline was −0.80 ([−1.06; −0.55], *P*  < 0.001).

#### 3.3.2. IGF-I, Cytokines, and MMP

Circulating levels of IGF-I decreased on Day 7 compared to baseline and were slightly lower in the NMES group compared to the control group (−10.3 vs. −27.7, respectively; MD [95% CI]: −13.43 [−31.05; 4.19], *P*=0.07). MMP-2 increased slightly in the NMES group (+0.13) and decreased in the control group (−0.05) (MD [95% CI]: −0.021 [−0.022; 0.074], *P*=0.38). IL-4 (anti-inflammatory cytokine) levels and behavior were similar in both groups (MD [95% CI]: 0.16 [−0.14; 0.46], *P*=0.27) (Table 4).

Regarding pro-inflammatory cytokines, IFN-y was similar in both groups (MD [95% CI]: −0.05 [−1.21; 1.10], *P*=0.92) and TNF-*α* decreased slightly in the NMES group while remaining constant in the control group throughout the seven days (−0.3 vs. 0, respectively; MD [95% CI]: 0.28 [−0.778; 1.338], *P*=0.58) (Table 4).

Serum concentration of MMP-9 increased slightly in the NMES group (+0.13) and decreased in the control group (−0.05) (MD [95% CI]: −0.021[ −0.022; 0.074], *P*=0.65). No differences were found between groups for serum levels of IL-2 (*p*=0.29), IL-6 (*p*=0.78), or IL-10 (*p*=0.40). These data are summarized in Table 4.

Blood samples could not be collected on all days in six patients due to clinical reasons, such as anemia, coagulation disorder, or medical contraindication. Thirteen patients in the control group and 12 in the NMES group had valid blood samples that enabled the quantification of IGF-I and cytokines at all time points. Zymographic analyses for MMP-2 and MMP-9 were performed for nine patients.

#### 3.3.3. Clinical Outcomes

The following clinical outcomes were similar between the control and NMES groups (Table 2): days on invasive mechanical ventilation (9.9 ± 5.8 vs. 11.6 ± 5.0, respectively) (MD [95% CI]: −1.7 [−5.24; 1.74], *P*=0.31); ventilator-free days at 28 days (10.0 ± 8.6 vs. 8.6 ± 7.9, respectively) (MD [95% CI]: 1.35 [−3.95; 6.65], *P*=0.60); length of stay in ICU (16.1 ± 12.6 vs. 16.5 ± 11.7 (MD [95% CI]: −0.4 [−8.52; 7.60], *P*=0.90); length of stay in hospital (26.7 ± 25.1 vs. 25.5 ± 22.4, respectively) (MD [95% CI]: 1.1 [−14.76; 17.11], *P*=0.88); seven-day mortality (five vs. four patients, respectively); and 28-day mortality (seven vs. eight patients, respectively) (Table 2).

## 4. Discussion

Our results demonstrate that an early short-term NMES protocol is an effective strategy for maintaining muscle size and echogenicity as well as reducing the levels of signaling mediators of muscle growth and systemic inflammation in mechanically ventilated patients with traumatic brain injury (TBI).

Previous studies have demonstrated that NMES effectively attenuates the short-term loss of muscle size in critically ill patients [2, 26] and healthy individuals under cast immobilization [27]. However, the loss of muscle mass within the first week of intensive care due to septic shock was unaffected by similar electrical stimulation in one study [37]. In another study, an NMES protocol with a median duration of four days (range: two to 13 days) was ineffective at preserving muscle thickness in critically ill patients following cardiothoracic surgery [23]; in these patients, an increase in muscle thickness was only observed from the preoperative period to postoperative Day 1 (prior to randomization), which was attributed to surgically induced inflammation and positive intraoperative fluid balance. In the present study, we observed a gain in muscle thickness. After seven days of hospitalization, however, muscle thickness was significantly decreased in the control group compared to baseline, which did not occur in the patients subjected to electrical stimulation [38].

An increase in muscle echogenicity and decrease in thickness are associated with the loss of muscle function [14]. An early short-term NMES protocol effectively preserved muscle echogenicity in the present study, whereas a significant increase was found in the control group. These results are consistent with a recent publication that demonstrated the effect of NMES on muscle architecture, with the attenuation of muscle atrophy, neurophysiological disorders, and weakness [2].

A positive fluid balance and subsequent muscle edema due to capillary leakage also play a critical role in increasing echogenicity. However, the lack of a correlation between fluid balance and echogenicity suggests objective muscle impairment related to immobility [27]. In critically ill patients with severe sepsis, muscle echogenicity increased from Day 4 to Day 14, with a decreasing fluid balance on Day 14 and specific structural damage in muscle tissue (fibrosis and fatty infiltration) demonstrated by magnetic resonance imaging [39].

### 4.1. NMES Muscle Thickness and Echogenicity Preservation Mechanism

Short-term disuse atrophy has been primarily attributed to declines in muscle protein synthesis and induced anabolic resistance to protein ingestion [40]. Moreover, the daily decline in the muscle fiber cross-sectional area is greater in comatose patients than healthy subjects [41], confirming that the mechanisms implicated in muscle wasting in critically ill patients cannot be attributed merely to muscle disuse. Other factors linked to muscle proteolysis in critically ill patients are mechanical ventilation, glucocorticoid treatment, inflammation due to sepsis and polyneuropathy, and a higher metabolic demand [42].

The prevention of muscle wasting by NMES in the present study may be attributed to both the maintenance of muscle protein synthesis and the suppression of muscle protein breakdown [19, 38, 43]. The stimulated patients presented a lower decrease in IGF-I levels than the controls, suggesting that the anabolic pathway of muscle growth was preserved. The lack of mechanical load combined with increased levels of IL-6 reduces serum levels of IGF-I binding protein-3, thereby promoting the degradation of serum IGF-I, which lessens the effect of IGF-I on muscle growth [44]. As the ability of active strengthening exercises to induce muscle growth by impacting these molecular pathways has been suggested [45], NMES could have exerted a similar effect on the muscles [41]. A shift in the cytokine profile after NMES [46] similar to that found with active exercise has been demonstrated in healthy individuals [47].

Furthermore, patients with TBI exhibit an inflammatory state imbalance while still at the scene of trauma (prior to hospital admission) that persists in the first week of hospitalization [19]. Inflammation plays an important role in muscle degradation in critical illness myopathy [38, 43]. Although the sample was possibly insufficient to demonstrate the potential anti-inflammatory effects of NMES treatment via the modulation in the cytokine profile, the clinical effect was clearly noticeable, mainly after Day 5, with a greater anti-inflammatory profile in the patients subjected to NMES. Accordingly, NMES may have facilitated a favorable balance between pro- and anti-inflammatory cytokines, possibly due to direct stimulation of the muscle fibers. Pro-inflammatory TNF-*α* was decreased, whereas IFN-y presented a greater increase in patients following the NMES protocol. These changes may have promoted an anti-inflammatory environment via paracrine and endocrine effects, suggesting lower activation of proteolysis in the muscle cells [45].

MMP-9 mediates blood-brain barrier breakdown and is pro-inflammatory [48]. Lower plasma MMP-9 concentrations in the first 48 hours after injury predict a shorter ICU stay and lower mortality rate after severe trauma [49]. In the present study, NMES effectively reduced MMP-9 activity in mechanically ventilated trauma patients, although this did not translate into a shorter length of stay or lower mortality rate. The observed slower decrease in IGF-I and MMP-2 expression may indicate a lower impact of bed rest and critical illness on anabolic muscle synthesis pathways. The changes in cytokine levels and MMP-9 expression suggest that NMES creates a more favorable pro- and anti-inflammatory balance, minimizing muscle breakdown mediated by the inflammatory process.

### 4.2. Early Intervention

Our study confirms that early intervention decreases the loss of muscle mass and the inflammatory process, with a protective effect on muscle thickness and echogenicity [29]. With the proposed intervention protocol, NMES was initiated in the first 24 hours after hospital admission while the patient was still in the emergency room, which may have enhanced the effectiveness at impeding muscle wasting. In a previous study, “early” intervention involved the NMES protocol 2.5 to 4.6 days after the admission to the ICU [8]. According to Hodgson et al. [50], when interventions are truly started early (e.g., within 48 hours since the onset of critical illness and immobility), the focus of physical therapy and mobilization is on maintaining muscle mass and function.

Lastly, some difficulties in applying NMES in critically ill patients have previously been reported [29], mainly due to increased skin and soft tissue impedance and edema, which were not observed in our patients. Despite the positive fluid balance, which is generally associated with a reduction in neuromuscular excitability, this limitation could be overcome by using an adequate pulse duration according to a previous chronaxie assessment [12, 51], providing adequate NMES with efficient muscle contractions and no adverse effects.

### 4.3. Study Limitations

There are some limitations to the present study. First, muscle thickness (MT) has not been proven to be directly associated with the cross-sectional area (CSA) measure, which is associated with the loss of strength. However, MT is appropriate for monitoring muscle loss in ICU patients, especially when combined with another ultrasonographic measure (e.g., echogenicity) [15, 52]. Second, although NMES was a practical, feasible countermeasure for muscle wasting in mechanically ventilated patients with TBI, the maintenance of muscle thickness (MT) and echogenicity did not translate into clinical benefits, such as an increased survival rate or shorter hospital stay. However, clinical outcomes were only evaluated for exploratory purposes and for a more accurate description of the patients. Third, it was not possible to assess voluntary muscle strength or functional status due to the patients' level of consciousness. Future studies should be designed to evaluate the effectiveness of an early short-term NMES protocol considering additional long-term outcomes with assessments in the ICU and at hospital discharge and determine whether the return to baseline function occurs more quickly.

## 5. Conclusions

Our study provides evidence that an early short-term NMES protocol is a beneficial approach to preserving muscle echogenicity and thickness in mechanically ventilated patients with TBI as well as maintaining systemic muscle growth mediators and the inflammatory response in this population. NMES is a feasible intervention for preventing skeletal muscle atrophy in critically ill patients with TBI who cannot engage in conventional active mobility programs.

## Figures and Tables

**Table 1:** Clinical characteristics on admission in the control and NMES groups.

Characteristics	Control (*n* = 20)	NMES (*n* = 20)
Age (yr)	36.5 (13.5)	34.7 (11.2)
Male sex, *n* (%)	16 (80%)	16 (80%)
APACHE II score	16.7 (4.5)	16.1 (4.6)
AIS (head)	5 [5-5]	5 [5-5]
AIS (lower extremities)	1 [0-1]	1 [0-1]
Hemoglobin (g/dL)	12.0 (2.6)	12.6 (3.5)
Leukocytes (×10^3^/*μ*L)	13.875 (6.329)	16.552 (6.614)
PaO_2_/FiO_2_ ratio	258.7 (81.9)	282.5 (64.1)
ISS	30 [25–35]	30 [27–35]
Cause of injury		
Motorcycle, *n* (%)	3 (15%)	5 (25%)
Motor vehicle, *n* (%)	6 (30%)	1 (5%)
Beating, *n* (%)	3 (15%)	3 (15%)
Gunshot, *n* (%)	4 (20%)	2 (10%)
Pedestrians, *n* (%)	3 (15%)	8 (40%)
Fall, *n* (%)	1 (5%)	1 (5%)
Penetrating trauma mechanism, *n* (%)	5 (25%)	2 (10%)
Operative intervention, *n* (%)	13 (65%)	12 (60%)

Continuous variables are expressed as mean (standard deviation) or median [interquartile range], and categorical variables are expressed as absolute and relative value = *n* (%). NMES: neuromuscular electrical stimulation; APACHE II: Acute Physiology and Chronic Health Evaluation II; AIS: Abbreviated Injury Scale; ISS: Injury Severity Score; PaO_2_/FiO_2_: ratio of arterial oxygen tension to inspired oxygen fraction.

**Table 2:** Clinical outcomes in the control and NMES groups.

	Control (*n* = 20)	NMES (*n* = 20)	MD	[95% CI]	*p* value
Sepsis, *n* (%)	17 (85%)	16 (80%)	1		0.67
Septic shock, *n* (%)	9 (45%)	11 (55%)	2		0.52
Multi-organ failure, *n* (%)	4 (20%)	6 (30%)	2		0.46
Initiation of enteral nutrition, *d*	3.25 (1.88)	3.47 (2.03)	−0.22	[−1.49; 1.05]	0.72
Cumulative fluid balance 7 days, *L*	8.5 (6.9)	8.2 (4.2)	0.27	[−4.1; 4.7]	0.89
Days of sedation, *d*	7.6 (4.7)	8.8 (4.9)	−1.2	[−4.40; 1.91]	0.42
Time on invasive MV, *d*	9.9 (5.8)	11.6 (5.0)	−1.7	[−5.24; 1.74]	0.31
Ventilator-free days at 28 days, *d*	10 (8.6)	8.6 (7.9)	1.35	[−3.95; 6.65]	0.60
ICU length of stay, *d*	16.1 (12.6)	16.5 (11.7)	−0.4	[−8.52; 7.60]	0.90
Hospital length of stay, *d*	26.7 (25.1)	25.5 (22.4)	1.1	[−14.76; 17.11]	0.88
7-day mortality, *n* (%)	5 (25%)	4 (20%)	1		0.70
28-day mortality, *n* (%)	7 (35%)	8 (40%)	1		0.74

Continuous variables are expressed as mean (standard deviation), and categorical variables are expressed as absolute and relative value = *n* (%). MD: mean difference between groups and [95% CI]: 95% confidence interval; NMES: neuromuscular electrical stimulation; MV: mechanical ventilation; ICU: intensive care unit. Clinical outcomes between control and NMES were compared by non-paired *t*-test for continuous variable and chi-square test for dichotomous variable.

**Table 3:** Changes in muscle echogenicity and thickness of rectus femoris muscle at Day 7 from baseline.

	Control	NMES	Differential response
Echogenicity			
Baseline	63.9 (12.6)	64.7 (9.2)	p=0.84
Change	29.9 (2.1)	3.0 (1.2)	*p* < 0.001
Delta unadjusted	26.9 [21.98; 31.78]		*p* < 0.001
Delta adjusted for baseline	26.7 [21.51; 31.82]		*p* < 0.001
Thickness			
Baseline	4.23 (0.91)	4.36 (0.68)	*p*=0.65
Change	−0.79 (0.12)	−0.01 (0.06)	*p* < 0.001
Delta unadjusted	−0.78 [−1.05; −0.51]		*p* < 0.001
Delta adjusted for baseline	−0.80 [−1.06; −0.55]		*p* < 0.001

Continuous variables are expressed as mean (standard deviation). Change and delta are expressed as mean difference [95% confidence interval]. Control: control group; NMES: neuromuscular electrical stimulation group. Data were assessed by non-paired *t*-student test and changes in muscle echogenicity and thickness from baseline to Day 7 were assessed using unadjusted and adjusted regression analysis.

**Table 4:** IGF-I, cytokines, and MMP over the first seven days after hospital admission in control (*n* = 13) and NMES (*n* = 12) groups.

	Day 1	Day 2	Day 3	Day 4	Day 5	Day 7	MD [95% CI] - Difference from D7 to D1
IGF-I, (ng/mL)							
Control	60.9 (28.0)	47.7 (24.5)	43.7 (26.7)	42.9 (17.8)	41.7 (16.3)	33.2 (15.5)	−13.43 [−31.05; 4.19]
NMES	55.8 (24.2)	44.5 (23.1)	42.2 (19.1)	40.8 (18.6)	43.3 (20.7)	45.5 (29.3)	
IL-2, (pg/mL)							
Control	8.3 (0.9)	8.5 (0.8)	8.6 (1.1)	8.8 (1.3)	8.8 (1.2)	8.7 (1.4)	0.36 [−0.34; 1.07]
NMES	8.2 (1.0)	8.7 (1.2)	8.6 (0.6)	8.1 (1.0)	8.0 (1.6)	7.9 (1.2)	
IL-4, (pg/mL)							
Control	4.1 (0.6)	3.9 (0.4)	3.8 (0.5)	4.3 (0.8)	4.3 (0.8)	4.2 (0.8)	0.16 [−0.14; 0.46]
NMES	4.0 (1.1)	3.9 (0.9)	3.8 (0.7)	4.1 (1.0)	3.6 (0.5)	4.3 (0.6)	
IL-6, (pg/mL)							
Control	231 (217)	312 (200)	289 (245)	253 (165)	236 (157)	203 (167)	−12.12 [−105.1; 80.84]
NMES	327 (228)	340 (247)	212 (95)	185 (116)	294 (212)	238 (172)	
IL-10, (pg/mL)							
Control	9.8 (7.6)	9.3 (5.3)	8.5 (6.8)	9.1 (6.2)	11.5 (8.5)	7.6 (4.3)	0.148 [−2.141; 5.120]
NMES	9.9 (6.8)	7.0 (3.7)	10.0 (5.9)	6.7 (3.6)	5.9 (2.3)	7.6 (4.2)	
TNF-*α*, (pg/mL)							
Control	2.8 (1.1)	2.8 (1.2)	2.8 (1.4)	2.8 (1.5)	3.0 (1.6)	2.8 (1.4)	0.28 [−0.77; 1.33]
NMES	2.7 (1.5)	2.7 (1.50)	2.6 (1.5)	2.7 (1.3)	2.2 (1.2)	2.4 (1.0)	
IFN-y, (pg/mL)							
Control	4.9 (1.4)	5.0 (1.3)	5.7 (1.6)	5.5 (1.6)	5.5 (1.3)	6.1 (1.4)	−0.05 [−1.21; 1.10]
NMES	5.0 (1.2)	5.4 (2.6)	6.0 (2.2)	5.6 (2.0)	5.2 (1.3)	5.8 (1.6)	
MMP-2, (active)							
Control (*n* = 5)	0.110 (0.026)	0.251 (0.110)	0.187 (0.034)	0.190 (0.041)	0.197 (0.032)	0.229 (0.057)	0.016 [−0.014; 0.046]
NMES (*n* = 4)	0.133 (0.055)	0.170 (0.016)	0.177 (0.032)	0.192 (0.033)	0.214 (0.094)	0.182 (0.015)	
MMP-9, (active)							
Control (*n* = 5)	0.303 (0.229)	0.237 (0.050)	0.226 (0.073)	0.212 (0.076	0.144 (0.019)	0.252 (0.039)	−0.021 [−0.022; 0.074]
NMES (*n* = 4)	0.133 (0.050)	0.198 (0.064)	0.198 (0.065)	0.193 (0.048)	0.217 (0.034)	0.269 (0.070)	

Data are expressed as mean ± standard deviation. MD: mean difference between groups and [95% CI]: 95% confidence interval of the difference among groups between Day 7 (D7) and Day 1 (D1). NMES: neuromuscular electrical stimulation.

## Data Availability

The data used to support the findings of this study are available from the corresponding author upon request.

## Abbreviations

NMES: Neuromuscular electrical stimulation
TBI: Traumatic brain injury
CONT: Control
IGF-I: Insulin-like growth factor I
MMP: Matrix metalloproteinases
IL: Interleukin
TNF: Tumor necrosis factor
IFN-y: Interferon gamma
ICU: Intensive care unit
AU: Arbitrary unit
SDS: Sodium dodecyl sulfate
APACHE II: Acute Physiology and Chronic Health Evaluation II
AIS: Abbreviated Injury Scale
ISS: Injury Severity Score
MT: Muscle thickness
CSA: Cross-sectional area.

## Additional Points

*Highlights.* (i) Early short-term (five days) NMES effectively preserves muscle echogenicity and thickness in mechanically ventilated patients with TBI. (ii) Early short-term (five days) NMES maintains muscle growth and inflammatory mediators in mechanically ventilated patients with TBI. (iii) NMES is a feasible intervention for critically ill patients with TBI who cannot engage in conventional active mobility programs.
